# Reveal key genes and factors affecting athletes performance in endurance sports using bioinformatic technologies

**DOI:** 10.1186/s12863-023-01106-9

**Published:** 2023-02-21

**Authors:** Juan Yan, Jie Bai

**Affiliations:** Woosuk University, 443 Samnye-Ro, Samrye-Eup, Wanju-Gun, Jeollabuk-Do, Wonju, Korea

**Keywords:** Bioinformatics, Microarray, Gene expression, Aerobic activity, Athlete

## Abstract

Medium-intensity activities comprise the major proportion of many sorts of sports. The energy consumption of athletes has been a research emphasis for the purpose of improving both training efficiency and competition performance. However, the evidence based on large-scale gene screen has been rarely performed. This is a bioinformatic study revealing the key factors contributed to the metabolic difference between subjects with different endurance activity capacities. A dataset comprised of high- (HCR) and low-capacity running (LCR) rats was used. Differentially expressed genes (DEGs) were identified and analysed. The Gene Ontology (GO) and Kyoto Encyclopaedia of Genes and Genomes (KEGG) pathway enrichment was obtained. The DEGs' protein–protein interaction (PPI) network was built, and the enriched terms of the PPI network were also analysed. Our findings showed that the GO terms were enriched in lipid metabolism-related terms. The KEGG signalling pathway analysis enriched in the ether lipid metabolism. Plb1, Acad1, Cd2bp2, and Pla2g7 were identified as the hub genes. This study provides a theoretical foundation showing lipid metabolism plays an important role in the performance of endurance activities. Plb1, Acad1, and Pla2g7 may be the key genes involved. The training plan and diet for athletes can be designed based on above results and expecting a better competitive performance.

**Supplementary Information:**

The online version contains supplementary material available at 10.1186/s12863-023-01106-9.

## Introduction

Competitive athletic specialities are highly involved in oxygen consumption and metabolism. The performance of athletes is relied on the metabolic capabilities. Different sports have noticeable difference in oxygen consumption patterns. Maximum Oxygen Consumption (VO2 Max, ml/min/kg) refers to the maximum oxygen consumption while an individual is under maximal exercise. It is an indicator reflecting cardiorespiratory fitness and endurance capacity in exercise performance. Mishra et al. indicated that the football players (67.6700) have the highest VO2 Max, followed by basketball (65.5550), volleyball (63.2667) and hockey players (62.3858) [1]. The tennis and badminton players have lower VO2 Max, which are 50.69 and 50.20 respectively [2]. Noticeably, although some moderate-intensity sports such as tennis and badminton show lower VO2 Max, they still reach the maximum fat oxidation ranging from 45 to 65% VO2 Max [3]. Additionally, a series of terms comprehensively evaluate the pulmonary function capacities, such as vital capacity (VC), forced vital capacity (FVC), forced expiratory volume in one second (FEV1), and maximum voluntary ventilation (MVV). VC and FVC are defined as the maximal inhalation/exhalation volume in either relaxed or forced manner, respectively. MVV is the maximal volume of air that an individual can inhale/exhale by voluntary effort in one minute. FEV1 represents the maximal amount of air can forcibly exhale during the first second after a maximal inhalation. They are important measurements of pulmonary function. Usually a higher value indicates a better pulmonary function, or from another perspective, a more intensive activity engagement. Mazic et al. [4] investigated the VC, FVC, FEV1, and MVV of 493 top athletes in 15 different sports disciplines. It was found that basketball players, water polo players, and rowers all exhibited better VC, FVC, FEV1 than healthy inactive controls. When compared to controls, football and volleyball players showed lower VC and FVC. Boxing, kayaking, rugby, handball, taekwondo, and tennis all had reduced peak expiratory flow. These results indicate that the performance of football, basketball, and volleyball could be the subjects that are influenced mostly by oxygen metabolic capability. Despite of not being included in these subjects, tennis was indicated in another research that the FVC, FEV1, and MVV ratios were higher in tennis players than in the sedentary control individuals [5]. Collectively, these evidence raise needs of understanding the metabolic mechanism of aerobic-relied sports, which could be beneficial to the training and competition. However, the key factors at gene level influencing athlete’s metabolism have been rarely studied. This bioinformatic study can help to reveal the key node of improving the metabolic capabilities and thus the performance. The results can be used to design a more scientific diet and training strategy.

High-capacity running (HCR) and low-capacity running (LCR) rats are an ideal animal model for studying the interaction between aerobic capacities and chronic disease. They are artificial selection for intrinsic aerobic endurance running capacity, which is described in detail in a previous report [6]. Briefly, the HCR lines have 171% longer maximal running distance until exhaustion than the LCR lines after six generations of selection.. In this bioinformatic study, HCR and LCR rats at generation 18 were adopted. We assume they have different gene expression levels which led to huge aerobic capacities between HCR and LCR rats.

Microarray technology provides a high throughput method that can analyse tens of thousands of gene expression patterns in one test [7]. It is a powerful tool for comparison of bulk gene expression between conditions. Open-source databases such as the Gene Expression Omnibus (GEO) database [8] can be used to upload and share microarray dataset. Kivelä et al. [9] were using HCR and LCR rat model to study connections between low aerobic exercise capacity and complex metabolic diseases. They briefly displayed a preliminary microarray screen for the genome expression profiles with aerobic endurance capacity and metabolic disease risk factors. This study was conducted based on selected Kivelä’s data on gene expression patterns affected by rat strains (HCR or LCR), and further analysed the in-depth understanding of key genes and signalling pathways involved in performance of aerobic exercise. Datasets were downloaded containing gene expression of HCR and LCR rats. Differentially expressed genes (DEGs) were identified, and the hub genes and critical terms involved were investigated using Gene Ontology (GO), Kyoto Encyclopaedia of Genes and Genomes (KEGG), and protein–protein interaction (PPI) network analyses. In summary, 31 DEGs and 4 hub genes were identified. The lipid metabolism may be a key factor in improving athlete’s performance in endurance exercise.

## Materials and methods

### Microarray data information

GSE17190 gene expression profiles were obtained using the platform Illumina ratRef-12 v1.0 expression beadchip (Illumina, San Diego, US) [9] from a public functional genomics data repository GEO database (https://www.ncbi.nlm.nih.gov/geo) [8]. To screen HCR and LCR rats, endurance running ability was measured on a treadmill, and the total distance covered throughout the test was utilised to calculate maximum aerobic exercise capacity. Rats with the maximum running capacity from each generation were bred together to create the HCR strain, whereas rats with the lowest running capacity were bred together to create the LCR strain. 24 female rats (12 HCR and 12 LCR) from generation 18 was used in the study. Detailed information about the animals can be found in the reference [9], and all webtools and software used in this study can be found in the Appendix.

### Data preprocessing and identification of DEGs

The preprocessing consists of several steps. In the raw data, the commercial probe serial numbers were converted into official gene symbols. Then uploaded onto the NetworkAnalyst 3.0 (https://www.networkanalyst.ca) [10], which is a visual analytics platform for comprehensive gene expression profiling and meta-analysis, for further preprocessing. Genes with variance percentile rank lower than 15, abundance lower than 4, or unannotated were discarded. After that, log2 transformation on the gene expression levels was performed for standardisation. The Limma package was then utilised to identify the up-regulated and down-regulated DEGs between HCR and LCR rats. The Benjamini–Hochberg test was also used to adjust the p-value. Finally, the DEG cut-off criterion was set to log2 fold change |log2FC|> 0.5 with an adjusted *P* < 0.05.

### GO and pathway enrichment analyses

The g:Profiler (http://biit.cs.ut.ee/gprofiler/) is a publicly accessible web service for analysing and altering gene lists derived from high-throughput genomic data [11]. The g:GOSt on g:Profiler was used to do GO enrichment and KEGG pathway enrichment analysis (based on KEGG Biological Pathway database [12]) of DEGs in this study. *P* < 0.05 and the custom-made g:SCS algorithm [11] were used as cut-off criterion. This combination gives a tighter threshold to significant results hence more reliable, as it considers the set structure underlying gene sets annotated to terms of each organism [11]. Biological Processes (BP), Cellular Component (CC), and Molecular Function (MF) are all included in the GO analysis.

### PPI network construction

The STRING (https://string-db.org) database [13] was used to recover the predicted associations between proteins encoded by DEGs and other proteins in order to understand the molecule mechanism and study the interactions between dynamic compression and chondrogenesis, as well as between proteins encoded by DEGs and other proteins. Since the number of identified DEGs is relatively low, PPI significance is defined as a confidence score of > 0.4, which is a range of medium to high confidence providing a balance between false positive and missing information. The false positive results can be identified by the following wet lab experiments. To visualise a PPI network, the interaction data was uploaded into the Cytoscape programme (version 3.8.0). In the PPI network, nodes represent proteins, and edges represent interactions. Their interaction is constructed based on the identified biological facts stored in the STRING database. The isolated proteins were excluded and the main network was preserved. The degree distribution was calculated by calculating the number of connections between the network's distinct proteins.

### PPI network function analysis

The g:Profiler was used to further analyse GO terms and pathway enrichment in the main PPI network found by the Cytoscape. This can provide a more accurate information about the function of key genes.

## Results

### Data preprocessing and identification of DEGs

**Fig. 1:**
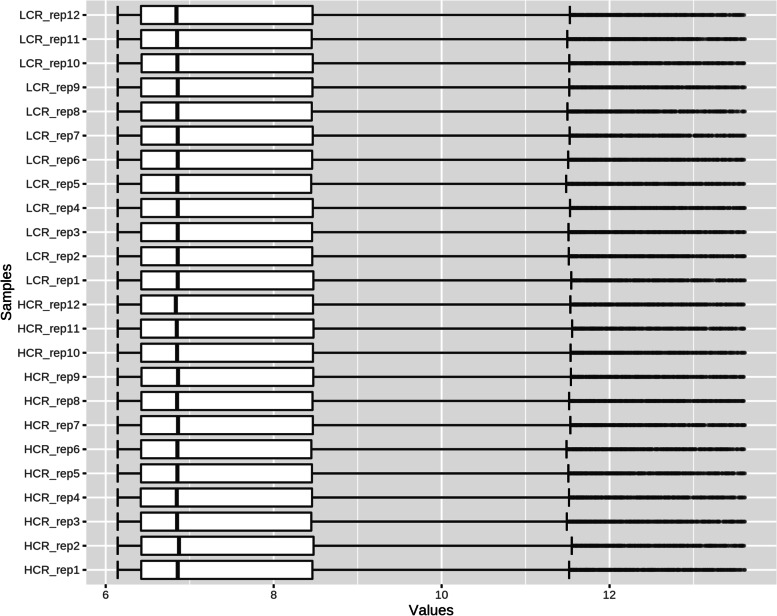
This is a figure. Schemes follow the same formatting

**Fig. 2:**
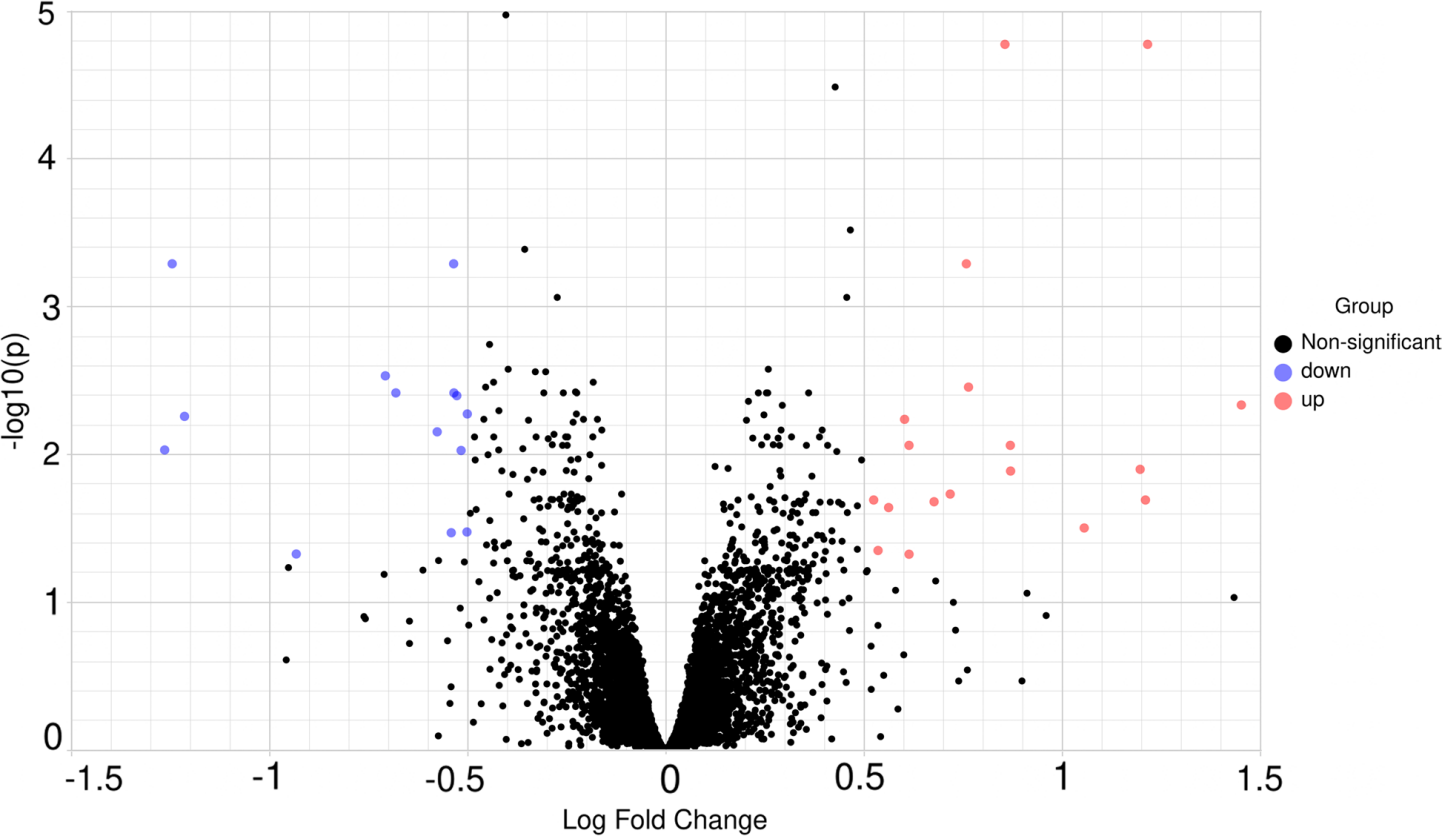
All genes found in the microarray are shown as a volcano. A gene is represented by each dot. Down- (blue) and up-regulated genes (red) are distinguished by colours. The log2-base fold change is on the X-axis, while the log10-base corrected P-value is on the Y-axis

**Fig. 3:**
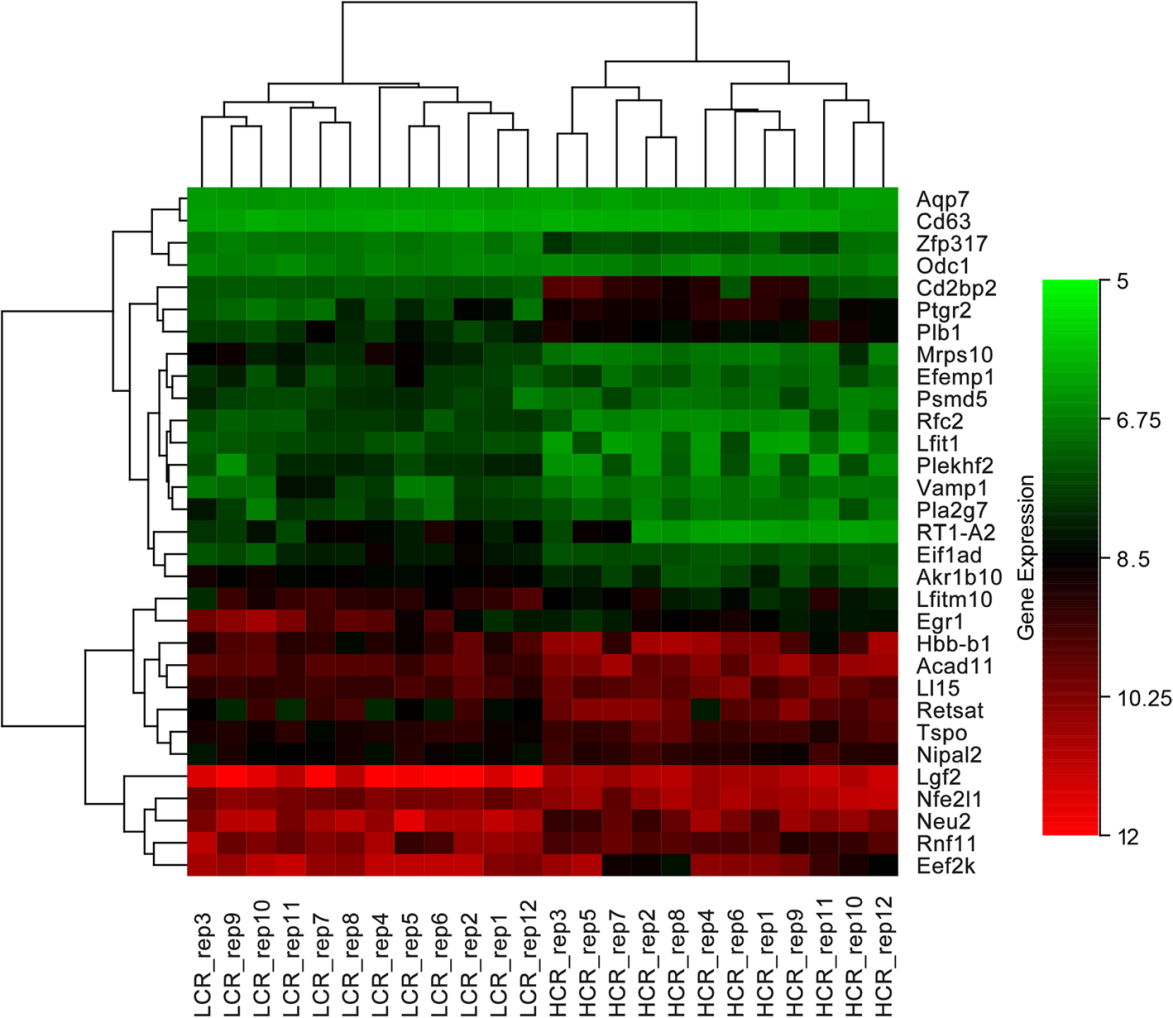
Cluster heatmap depicts DEG-based hierarchical clustering analysis findings. Each column is a sample, and each row represents a DEG. The colour represents the relative degree of gene expression (log2 transformed). Green denotes lower gene expression levels, whereas red suggests greater levels

After normalisation, the gene expression data is shown in Fig. 1. In HCR rats, a total of 31 DEGs were identified, with 17 (54.84%) up-regulated genes and 14 (45.16%) down-regulated genes compared to LCR rats. The volcano map (Fig. 2) shows the differential expression status of all discovered genes while highlighting DEGs that are greater than the chosen cut-off value. Figure 3 shows the cluster heatmap of DGEs. To cluster the genes and generate the dendrograms, Euclidean distance was used. There are significant changes in DEG expression between the HCR and LCR groups, indicating that the DEGs are trustworthy and appropriate for further investigation. Table 1 lists the top ten most substantially up-regulated and down-regulated genes.

**Table 1:** The top 10 most significantly up-regulated and down-regulated DEGs

Up-regulated DEGs	Log2FC	*P*-value	Down-regulated DEGs	Log2FC	*P*-value	
*RT1-A2*	1.452	1.25 × 10^–5^	***Cd2bp2***	-1.2635		5.58 × 10^–5^
*Mrps10*	1.2156	2.97 × 10^–9^	***Ptgr2***	-1.2445		2.84 × 10^–7^
*Eef2k*	1.2102	1.92 × 10^–4^	***Retsat***	-1.2134		1.75 × 10^–5^
*Igf2*	1.1968	8.76 × 10^–5^	***Hbb-b1***	-0.9316		8.96 × 10^–4^
*Egr1*	1.0557	4.32 × 10^–4^	***Acad11***	-0.70689		3.85 × 10^–6^
*Neu2*	0.87004	9.49 × 10^–5^	***Plb1***	-0.68037		7.22 × 10^–6^
*Plekhf2*	0.8694	4.88 × 10^–5^	***Nfe2l1***	-0.57617		2.82 × 10^–5^
*Akr1b10*	0.85584	3.88 × 10^–9^	***Aqp7***	-0.5407		4.89 × 10^–4^
*Ifit1*	0.76393	5.62 × 10^–6^	***Zfp317***	-0.53468		3.56 × 10^–7^
*Rfc2*	0.75845	3.53 × 10^–7^	***Tspo***	-0.53396		9.32 × 10^–6^

### GO and Pathway Enrichment Analyses

**Fig. 4:**
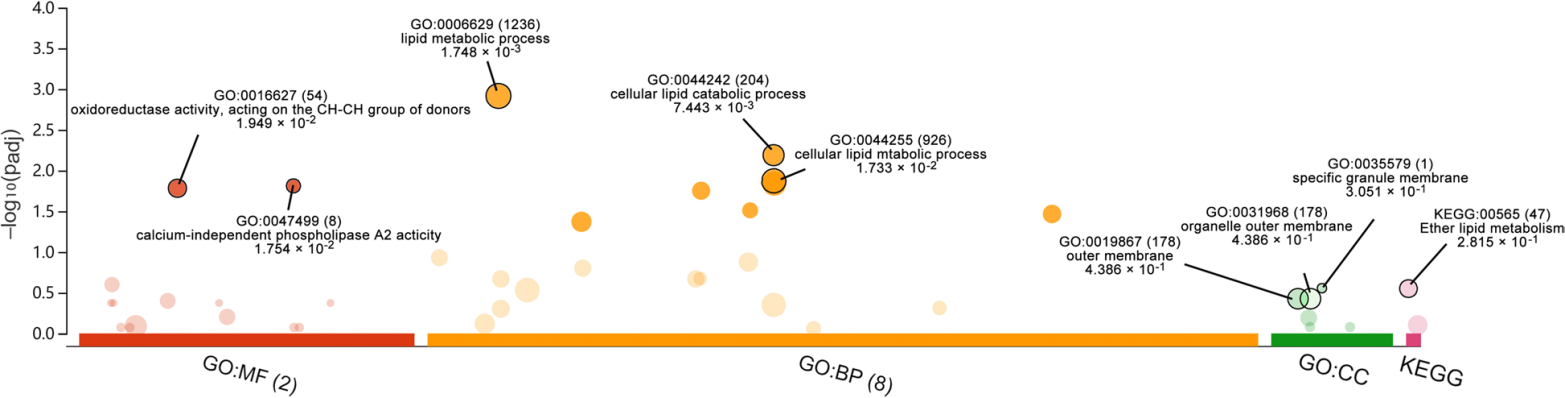
GO enrichment and KEGG pathway enrichment analysis. Each bubble represents a term. The height of the bubbles stands for the significance of enrichment. The horizontal distance stands for the similarity of term subtrees. Bubble size stands for term size (gene counts). The X-axis represents the group of functional terms and coloured by data sources, and the Y-axis lays out adjusted p-value in negative log10 scale

The biological function of DEGs was determined using GO enrichment and KEGG pathway enrichment studies. Lipid metabolic process, cellular lipid catabolic process, and cellular lipid metabolic process were the biological process with the greatest significant enrichment in GO terms. The cellular component with the highest significant enrichment was specific granule membrane, organelle outer membrane, and outer membrane, although there was no obvious statistical difference shown. The greatest significant enrichment in molecular function was calcium-independent phospholipase A2 activity and oxidoreductase activity, acting on the CH-CH group of donors. The ether lipid metabolism was shown to be associated to the difference of aerobic exercise capability in the KEGG pathway enrichment, without statistical significance. Figure 4 and Table 2 show the whole list of enhanced GO terms and KEGG pathways.

**Table 2:** Significantly enriched GO terms of DEGs

Category	GO ID	Description	Gene Count	*P*-value
BP	GO:0006629	lipid metabolic process	10	1.75 × 10^–3^
BP	GO:0044242	cellular lipid catabolic process	5	7.44 × 10^–3^
BP	GO:0044255	cellular lipid metabolic process	8	1.73 × 10^–2^
BP	GO:0034754	cellular hormone metabolic process	4	1.95 × 10^–2^
BP	GO:0044281	small molecule metabolic process	10	2.06 × 10^–2^
BP	GO:0042572	retinol metabolic process	3	3.26 × 10^–2^
BP	GO:0120254	olefinic compound metabolic process	4	3.76 × 10^–2^
BP	GO:0016042	lipid catabolic process	5	4.88 × 10^–2^
CC	GO:0035579	specific granule membrane	1	3.05 × 10^–1^
CC	GO:0031968	organelle outer membrane	3	4.39 × 10^–1^
CC	GO:0019867	outer membrane	3	4.39 × 10^–1^
MF	GO:0047499	calcium-independent phospholipase A2 activity	2	1.75 × 10^–2^
MF	GO:0016627	oxidoreductase activity, acting on the CH-CH group of donors	2	1.95 × 10^–2^
KEGG	KEGG:00565	Ether lipid metabolism	2	5.86 × 10^–4^

### PPI network construction

**Fig. 5:**
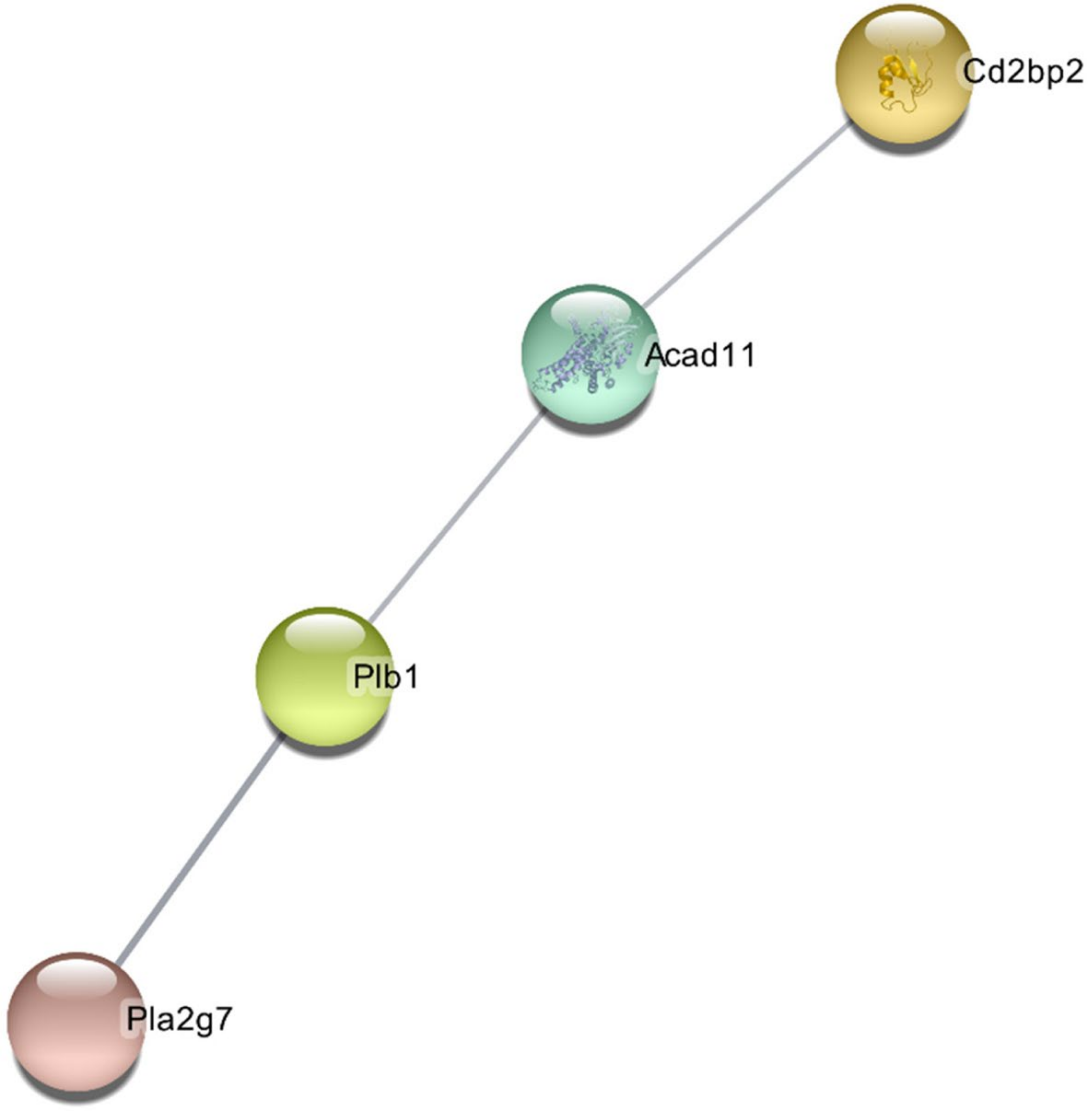
PPI network of all DEGs. In the main network, the degree of Plb1 and Acad11 were 2 and Cd2bp2 and Pla2g7 were 1

The STRING database created a PPI network comprising all DEGs (Fig. 5) with 4 nodes and 3 edges. The majority of proteins were isolated and thus excluded from the network. The Plb1, Acad1, Cd2bp2, and Pla2g7 were screened as hub genes. The degree of Plb1 and Acad11 were 2 and Cd2bp2 and Pla2g7 were 1. Plb1, Acad1, Cd2bp2, and Pla2g7 play a significant role in duration of aerobatic exercise.

### PPI network function analysis

**Fig. 6:**
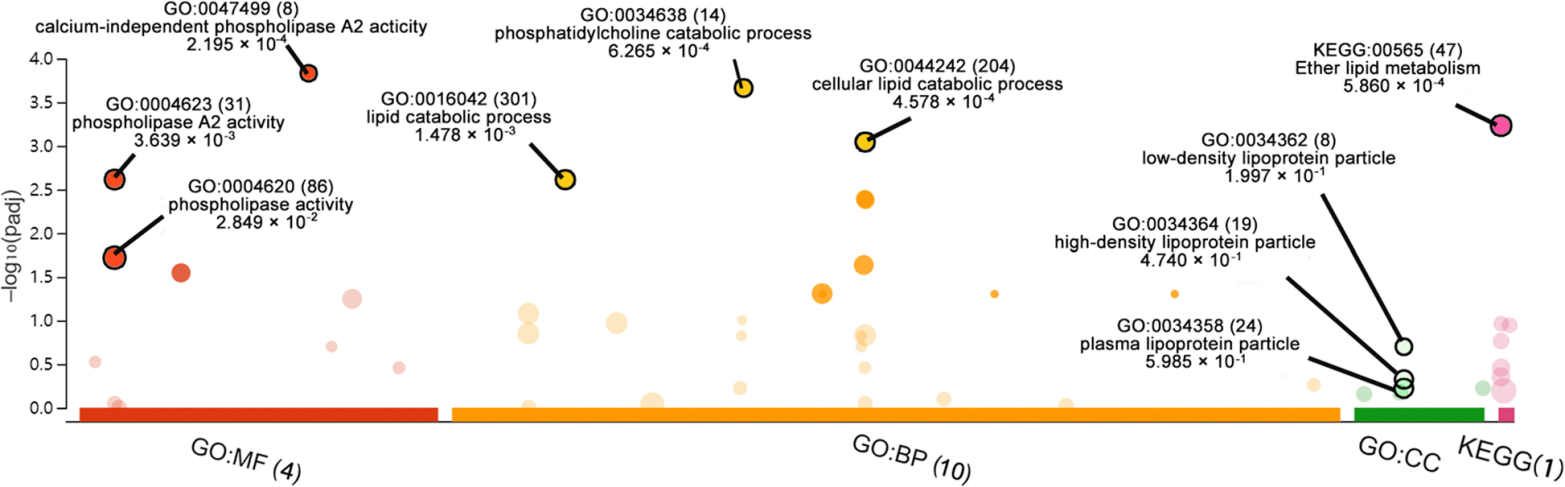
GO enrichment and KEGG pathway enrichment analysis of PPI main network. Each bubble represents a term. The height of the bubbles stands for the significance of enrichment. The horizontal distance stands for the similarity of term subtrees. Bubble size stands for term size (gene counts). The X-axis represents the group of functional terms and coloured by data sources, and the Y-axis lays out adjusted p-value in negative log10 scale

The Fig. 6 and Table 3 summarised the GO terms and KEGG pathway enriched in the PPI main network. The biological process with the most significant enrichment in GO terms were cellular lipid catabolic process, phosphatidylcholine catabolic process, and lipid metabolic process. In cellular component, low-density lipoprotein particle, high-density lipoprotein particle, and plasma lipoprotein particle were identified without significant statistical difference. In terms of molecular function, the most significant enrichment was calcium-independent phospholipase A2 activity, phospholipase A2 activity, and phospholipase activity. The ether lipid metabolism was enriched in the KEGG pathway enrichment (Fig. 6 and Table 3).

**Table 3:** Significantly enriched GO terms of PPI main network

Category	GO ID	Description	Gene Count	*P*-value
BP	GO:0044242	cellular lipid catabolic process	3	4.58 × 10^–4^
BP	GO:0034638	phosphatidylcholine catabolic process	2	6.27 × 10^–4^
BP	GO:0016042	lipid catabolic process	3	1.48 × 10^–3^
BP	GO:0046475	glycerophospholipid catabolic process	2	2.60 × 10^–3^
BP	GO:0009395	phospholipid catabolic process	2	7.12 × 10^–3^
BP	GO:0046470	phosphatidylcholine metabolic process	2	1.18 × 10^–2^
BP	GO:0046503	glycerolipid catabolic process	2	1.22 × 10^–2^
BP	GO:0034440	lipid oxidation	3	3.75 × 10^–2^
BP	GO:0044255	cellular lipid metabolic process	3	4.33 × 10^–2^
CC	GO:0034362	low-density lipoprotein particle	1	2.00 × 10^–1^
CC	GO:0034364	high-density lipoprotein particle	1	4.74 × 10^–1^
CC	GO:0034358	plasma lipoprotein particle	1	6.88 × 10^–1^
MF	GO:0047499	calcium-independent phospholipase A2 activity	2	2.20 × 10^–4^
MF	GO:0004623	phospholipase A2 activity	2	3.64 × 10^–3^
MF	GO:0004620	phospholipase activity	2	2.85 × 10^–2^
MF	GO:0016298	lipase activity	2	4.25 × 10^–2^
KEGG	KEGG:00565	Ether lipid metabolism	2	5.86 × 10^–4^

## Discussion

Competitive athletic specialities are facing huge energy consumption during training and competition. Carbohydrate and fat contributions to energy generation vary depending on exercise duration and intensity, training state, gender, and so forth. Football, basketball, volleyball, tennis, badminton, and others has traditionally been thought of being an intermittent activity, with bursts of high intensity movement interspersed with extended periods of medium-intensity activities (e.g. walking, jogging, etc.). Thus, professional players must therefore improve both anaerobic and aerobic capacities in order to prepare for competition. However, the medium-intensity activities occupy the majority time in a competitive game, player’s frequent movement ensures a good location for performing the last critical high intensity skills. This leads to the requirement on understanding the key factors improving the capability of endurance exercise. The evidence at gene level can be fundamental to provide an insight to metabolism and guidance to a better training/diet strategy.

This bioinformatic study found that compared to the LCR rats, the HCR rats had highly enriched GO terms regarding lipid metabolism, including lipid metabolic process, cellular lipid catabolic process, cellular lipid metabolic process, and phosphatidylcholine catabolic process. The KEGG signalling pathway analysis also showed the ether lipid metabolism, which is a lipid metabolism-related pathway, enriched in the HCR rats. Lipid is an important energy source during exercise. They are utilised in forms of circulating free fatty acids (FFAs) bound to albumin, triglycerides stored in very-low-density lipoprotein, and intramuscular triglyceride stores [14]. Exercise intensity influences substrate choice. Lipid is the primary fuel at low exercise intensities (under 40% VO2 Max), but as intensity increases, the body shifts to carbohydrate (CHO). Between 45 and 65% VO2 Max, maximum fat oxidation occurs and over this point the fuel preference balance switches toward carbohydrate. Endurance activities increase the energy utilisation from fat while sparing carbohydrate sources [3, 14]. A study investigated influence on plasma lipid parameters by high quality physical training in 11 groups of athletic specialities, including football, basketball, volleyball, boxing, wrestling, judo, sailing, skiing (slalom), track (two groups), throwing, and jumping. The results demonstrated that endurance sports, such as football, basketball, volleyball, short- and long-distance running showed higher high-density lipoprotein (HDL) and lipoprotein ratio factor (RF) values, compared to those strength sports such as wrestling, boxing, skiing (slalom), and throwing-jumping [15]. Another research studied 71 male athletes (25 football players, 14 volleyball players and 32 healthy sedentary subjects). Results showed that both football and volleyball players had considerably less body fat than the control group. Football players had the lowest body fat percentage [2]. Interestingly, there is evidence showing the capability of using lipid as energy is different in genders. A mice model study indicated that females had considerably increased gene expression in genes involved in skeletal muscle fatty acid oxidation. Female mice have a stronger endurance exercise capacity and a larger ability to mobilise and use fatty acids for energy [16]. However, we are unable to compare the gene expression difference in genders in this bioinformatic study as all rat participants were female. Collectively, the performance of aerobic exercise and training are highly relevant to the lipid metabolic ability and efficiency.

Plb1 is a protein coding gene and related to phospholipases and lipid metabolism. As FFAs are an importance energy source during exercise, a sufficient FFAs pool is critical for energy supply. Currently most of the reports of Plb1 focus on microbial species [17, 18]. Wright et al. [18] used yeast for studying FFA production. The yeast had high FFA production but the deletion of phospholipase genes Plb1 and Plb2 resulted in a 46% decrease in FFA levels and 105% increase in phospholipid levels. The decrease of FFA levels and increase of phospholipid levels strongly suggested that Plb1 is pivotal in FFAs formation and the FFAs were mainly generated through phospholipid hydrolysis [19]. However, it also indicated that the over expression of Plb1 didn’t witness a significant increase in FFAs, which suggested that phospholipase activity may not be a major limiting step in FFA production. There is also evidence showing the phospholipases are an important part of the virulence of pathogenic fungi [20]. So far, there lacks studies on mammals. Further research would help us understand the function of Plb1 in human and other animals.

Acad1 is also known as medium-chain acyl-CoA dehydrogenase (Acadm), an enzyme that catalyses the first step of β-oxidation and responsible for the breakdown of medium-chain fatty acids in the mitochondria [21]. Leg blood flow increases dramatically at the beginning of and throughout exercise, from around 0.3 L/min at rest to 5–6 L/min at moderate exercise intensities and up to 9 –12 L/min at 100% VO2 Max, which brings more FFAs and glucose [22]. Fatty acids are a primary source of energy for heart and oxidative skeletal muscle. Once within the myocyte, fatty acids are directed to either lipid metabolite production or mitochondrial β-oxidation. Intramuscular lipids can accumulate when fatty acid intake exceeds the rate of β-oxidation. On a mouse model, Acadm knockdown remarkably enhanced lipid accumulation [21]. In a study using biopsy specimens of 74 patients with nonalcoholic fatty liver disease (NAFLD), the expression of all genes (including Acad1) involved in the metabolism of fatty acids and iron were significantly downregulated as the stage and grade of NAFLD progressed [23]. It is shown that the Phosphatidylinositol 3-kinase (PI3K) signaling pathway plays an important role in the cellular lipid metabolism regulation. The PI3KR3 is a subunit of PI3K, it was increased after fasting and was downregulated in a high-fat diet (HFD) mouse model of fatty liver, which suggests a strong relationship between PI3KR3 and lipid metabolism. The overexpression of PIK3R3 upregulated the protein and mRNA expression levels of Acad1 and resulted in enhanced fatty acid β-oxidation and reduced fatty liver in vivo. Conversely, knockdown of PIK3R3 downregulated protein and mRNA expression levels of Acad1 which led to impaired hepatic fatty acid β-oxidation in cell culture and in vivo [24]. In terms of genders, it is shown that women have a lower respiratory exchange ratio (RER) compared with men, indicating higher lipid oxidation and can oxidize more fat during moderate intensity endurance exercise [25]. In summary, the Acad1 and PI3KR3 both are important candidates in lipid metabolism. They function in the body synergistically. A higher expression of these two genes may contribute to a more efficient energy gain from the lipocatabolic process.

There are limited reports on the function of Cd2bp2 and Pla2g7 in lipid metabolism. It has been known that the Cd2bp2 (adaptor protein CD2-binding protein 2) is a gene involved in oxidative phosphorylation, gluconeogenesis, lipid metabolism, signal transduction [26] and T-cell activation and immunology [27]. However, based on current literature, the role of Cd2bp2 largely remains unclear. Pla2g7 (platelet-activating factor acetylhydrolase) is well recognised for degrading platelet-activating factor, a phospholipid mediator of platelet aggregation and a powerful inflammatory stimulator and also a participant in lipid metabolism. It functions at the intersection of metabolism and immunity [28]. In human, Pla2g7 expression is increased in activated macrophages and atherosclerotic plaque foam cells [29]. It has also been identified as a key gene involved in coronary heart disease due to dysregulation of phospholipid metabolism [30]. In mice, obesity and a high-fat diet increased Pla2g7 expression, but caloric restriction reduced the expression. Pla2g7 regulates pathways that contribute to local and systemic metabolic integrity, immunological modulation, and inflammation through immunometabolic control in diverse tissues [28]. Similarly, another study reported the Pla2g7 had lower expression in humans undergoing caloric restriction (CR). The CR-induced low expression of Pla2g7 can decrease glucose consumption, lead to better adipose tissue metabolism, lower inflammation, and reduced thymic lipoatrophy [31]. The Cd2bp2 and Pla2g7 may be two new interesting targets and worthy of further investigations.

Based on above results, lipid metabolism is a critical point to the athletes participating in endurance exercise. We raise suggestions for improving their performance. The fat, in the form of our bodily reserves, provides a virtually limitless supply of energy. Improving our capacity to transfer it into the muscle and oxidise it during activity is a vital adaptation to training. Even the most highly trained athletes have not reached their maximum fat oxidation capacity during exercise, which may be improved even more by eating a high-fat meal before to the activity. Although fat oxidation has limited capability as a fuel source for high-intensity activities, if fat supplements or other items could improve fat utilisation at more moderate exercise intensities, it might provide a mechanism to 'spare' muscle glycogen reserves for the high-intensity stages of sport. Research suggests that diets containing 32% to 55% fat can boost endurance capacity compared to diets containing 15% fat [32]. Another strategy is 'fat adaptation' diet, a diet in which well-trained endurance athletes take a high-fat, low-CHO diet for up to 2 weeks while continuing their regular training, followed by CHO restoration (consuming a high-CHO diet and tapering for 1 – 3 days before a big endurance event). This "dietary periodization" approach boosts whole-body and muscle fat oxidation while decreasing muscle glycogenolysis during submaximal exercise as compared to an isoenergetic CHO diet over the same intervention period [33]. Except from diet, scientific training methods are also indispensable. Besides, the difference of gene expression levels is non-negligible. It could potentially affect the capability of lipid metabolism of athletes. Those athletes who innately have higher expression level of Plb1 and Acad1 and lower level of Pla2g7 may have more efficient lipid energy usage ability and thus better performance in endurance exercise. The role of Cd2bp2 is worthy to be further investigated.

Although this study has identified some key factors and genes affecting the performance in endurance exercise through bioinformatic methods, there still are some limitations. Above findings are database and algorism-based, they have not been verified by lab-based verification experiment. The bioinformatic tools are largely reliable but few false positive or false negative results may be inevitable due to the chosen thresholds. We are planning a following verification experiment in the near future to further support our findings. Additionally, wild type control groups are missing, as they are not included in the original dataset. However, the bioinformatic databases are running in a manner of voluntary sharing, limited datasets match our criteria. For example, some animals were treated with drugs or underwent extra conditions. The dataset utilised in this manuscript is the most matching one, despite it is not the very latest one. Rats have genetic similarity to human beings, but they still could lead to different results in the same condition as human. Using rats instead of human for study is a consideration of ethics and expense. The results we have in this study may not fully represent those of human beings.

## Conclusions

In this study we used a set of gene microarray data to identify the RNA expression difference between HCR and LCR rats. It was found that the major difference in biological process is the HCR rats has better lipid metabolic capabilities, which may result in better efficiency of energy gain than the LCR rats. The KEGG signalling pathway analysis enriched in the ether lipid metabolism. Four hub genes including Plb1, Acad1, Cd2bp2, and Pla2g7 were identified. We suggest a diet strategy contains sufficient fat intake can improve the performance in endurance exercise by maximising the usage of lipid energy. Innate difference of gene expression levels may also affect the performance of athletes.

## Supplementary Information

**Additional file1:** **Figure 1. **This is a figure. Schemes follow the same formatting. **Figure 2. **All genes found in the microarray are shown as a volcano. A gene is represented by each dot. Down- (blue) and up-regulated genes (red) are distinguished by colours. The log2-base fold change is on the X-axis, while the log10-base corrected P-value is on the Y-axis.** Figure 3. **Cluster heatmap depicts DEG-based hierarchical clustering analysis findings. Each column is a sample, and each row represents a DEG. The colour represents the relative degree of gene expression (log2 transformed). Green denotes lower gene expression levels, whereas red suggests greater levels. **Figure 4. **GO enrichment and KEGG pathway enrichment analysis. Each bubble represents a term. The height of the bubbles stands for the significance of enrichment. The horizontal distance stands for the similarity of term subtrees. Bubble size stands for term size (gene counts). The X-axis represents the group of functional terms and coloured by data sources, and the Y-axis lays out adjusted p-value in negative log10 scale.** Figure 5. **PPI network of all DEGs. In the main network, the degree of Plb1 and Acad11 were 2 and Cd2bp2 and Pla2g7 were 1.** Figure 6. **GO enrichment and KEGG pathway enrichment analysis of PPI main network. Each bubble represents a term. The height of the bubbles stands for the significance of enrichment. The horizontal distance stands for the similarity of term subtrees. Bubble size stands for term size (gene counts). The X-axis represents the group of functional terms and coloured by data sources, and the Y-axis lays out adjusted p-value in negative log10 scale.

## Data Availability

Dataset GSE17190 used in this study can be downloaded on the GEO database (https://www.ncbi.nlm.nih.gov/geo/query/acc.cgi?acc=GSE17190).
