# Supplementary material for: Reveal key genes and factors affecting athletes performance in endurance sports using bioinformatic technologies
Source: BMC Genom Data. 2023 Feb 21;24:10. doi: 10.1186/s12863-023-01106-9 (PMC9945390; doi:10.1186/s12863-023-01106-9)
Supplement: Supplementary file 1 — Additional file1: Figure 1. This is a figure. Schemes follow the same formatting. Figure 2. All genes found in the microarray are shown as a volcano. A gene is represented by each dot. Down- (blue) and up-regulated genes (red) are distinguished by colours. The log2-base fold change is on the X-axis, while the log10-base corrected P-value is on the Y-axis. Figure 3. Cluster heatmap depicts DEG-based hierarchical clustering analysis findings. Each column is a sample, and each row represents a DEG. The colour represents the relative degree of gene expression (log2 transformed). Green denotes lower gene expression levels, whereas red suggests greater levels. Figure 4. GO enrichment and KEGG pathway enrichment analysis. Each bubble represents a term. The height of the bubbles stands for the significance of enrichment. The horizontal distance stands for the similarity of term subtrees. Bubble size stands for term size (gene counts). The X-axis represents the group of functional terms and coloured by data sources, and the Y-axis lays out adjusted p-value in negative log10 scale. Figure 5. PPI network of all DEGs. In the main network, the degree of Plb1 and Acad11 were 2 and Cd2bp2 and Pla2g7 were 1. Figure 6. GO enrichment and KEGG pathway enrichment analysis of PPI main network. Each bubble represents a term. The height of the bubbles stands for the significance of enrichment. The horizontal distance stands for the similarity of term subtrees. Bubble size stands for term size (gene counts). The X-axis represents the group of functional terms and coloured by data sources, and the Y-axis lays out adjusted p-value in negative log10 scale. [file 12863_2023_1106_MOESM1_ESM.docx]

Reveal Key Genes and Factors Affecting Athletes Performance in Endurance Sports Using Bioinformatic Technologies

Juan Yan ^1^, and Jie Bai ^1,^*

^1^ Woosuk University, 443 Samnye-ro, Samrye-eup, Wanju-gun, Jeollabuk-do, Korea; yanjuan@stu.woosuk.ac.kr

***** Correspondence: baijie1981@stu.woosuk.ac.kr

**Appendix Table.** Webtools and software used in this study.

| **Software/website** | **Website address** |
| --- | --- |
| GEO database | https://www.ncbi.nlm.nih.gov/geo |
| NetworkAnalyst 3.0 | https://www.networkanalyst.ca |
| g:Profiler | http://biit.cs.ut.ee/gprofiler/ |
| STRING database | https://string-db.org |
| Cytoscape software (version 3.8.0) | https://cytoscape.org |
